# SUMOylation in Human Pathogenic Fungi: Role in Physiology and Virulence

**DOI:** 10.3390/jof6010032

**Published:** 2020-03-04

**Authors:** Mahima Sagar Sahu, Sandip Patra, Kundan Kumar, Rupinder Kaur

**Affiliations:** 1 Laboratory of Fungal Pathogenesis, Centre for DNA Fingerprinting and Diagnostics, Hyderabad 500039, Telangana, India; mahimasagar@cdfd.org.in (M.S.S.); sandippatra@cdfd.org.in (S.P.); kundankumar@cdfd.org.in (K.K.); 2 Graduate studies, Regional Centre for Biotechnology, Faridabad 121001, Haryana, India; 3 Graduate studies, Manipal Academy of Higher Education, Manipal 576104, Karnataka, India

**Keywords:** small ubiquitin-like modifier (SUMO), human pathogenic fungi, *Candida glabrata*, *Candida albicans*, SUMO-specific proteases and ligases, virulence, stress survival

## Abstract

The small ubiquitin-related modifier (SUMO) protein is an important component of the post-translational protein modification systems in eukaryotic cells. It is known to modify hundreds of proteins involved in diverse cellular processes, ranging from nuclear pore dynamics to signal transduction pathways. Owing to its reversible nature, the SUMO-conjugation of proteins (SUMOylation) holds a prominent place among mechanisms that regulate the functions of a wide array of cellular proteins. The dysfunctional SUMOylation system has been associated with many human diseases, including neurodegenerative and autoimmune disorders. Furthermore, the non-pathogenic yeast *Saccharomyces cerevisiae* has served as an excellent model to advance our understanding of enzymes involved in SUMOylation and proteins modified by SUMOylation. Taking advantage of the tools and knowledge obtained from the *S. cerevisiae* SUMOylation system, research on fungal SUMOylation is beginning to gather pace, and new insights into the role of SUMOylation in the pathobiology of medically important fungi are emerging. Here, we summarize the known information on components of the SUMOylation machinery, and consequences of overexpression or deletion of these components in the human pathogenic fungi, with major focus on two prevalent *Candida* bloodstream pathogens, *C. albicans* and *C. glabrata*. Additionally, we have identified SUMOylation components, through in silico analysis, in four medically relevant fungi, and compared their sequence similarity with *S. cerevisiae* counterparts. SUMOylation modulates the virulence of *C. albicans* and *C. glabrata*, while it is required for conidia production in *Aspergillus nidulans* and *A. flavus*. In addition to highlighting these recent developments, we discuss how SUMOylation fine tunes the expression of virulence factors, and influences survival of fungal cells under diverse stresses in vitro and in the mammalian host.

## 1. Introduction

**Figure 1:**
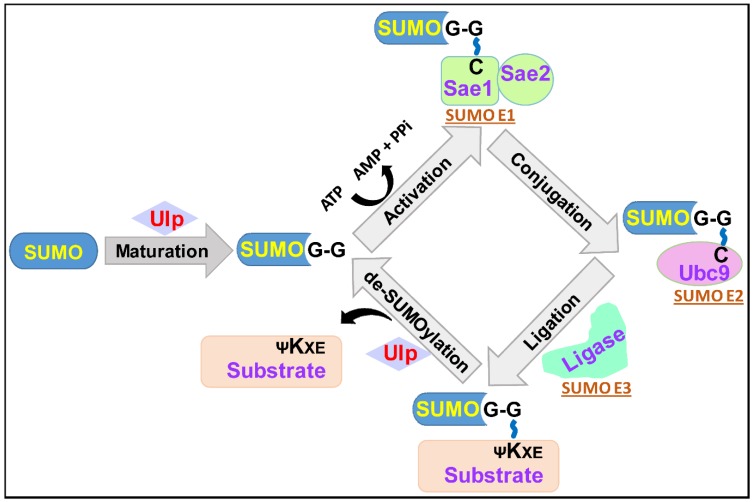
Schematic illustration of the steps involved in SUMO-conjugation and de-conjugation processes. SUMOylation starts with the action of SUMO-specific proteases (Ulp/SENP family) on newly synthesized SUMO, which leads to the generation of mature SUMO with exposed carboxyl-terminal GG motif. The second step involves SUMO-activating enzyme (E1)-mediated activation of the SUMO protein in an ATP-dependent fashion, by first inducing adenylation of the SUMO carboxyl-terminal, followed by the energy-rich thioester bond formation between the thiol group of cysteine present in the catalytic site of the E1 enzyme and the C-terminal glycine residue of the SUMO protein. The activated SUMO is next transferred from the E1 enzyme to the cysteine residue present in the catalytic site of the SUMO-conjugating enzyme (E2), through the thioester linkage. With the help of the SUMO ligase (E3), SUMO is further transferred from the E2 enzyme to the target protein via isopeptide bond formation between the C-terminal carboxyl group of SUMO and the ε-amino group of the lysine residue in the target protein. SUMO-specific proteases also cleave an isopeptide bond between SUMO and the target protein, resulting in the generation of an unSUMOylated target protein and free SUMO.

A reversible post-translational modification of proteins, mediated by a highly conserved small ubiquitin-related modifier (SUMO), regulates numerous physiological processes [1,2,3]. SUMO is a ∼11 kDa polypeptide, that is attached covalently, via an isopeptide bond, to the amino group of the lysine residue in cellular substrate proteins [1,4]. This conjugation is predominantly catalyzed by SUMO ligases, and is the fourth step in the process of SUMOylation [3,5]. The four enzymatic steps in the SUMOylation cascade consist of: (i) SUMO processing by SUMO-specific proteases to generate mature SUMO with an exposed carboxyl-terminal diglycine (GG) motif; (ii) formation of a thioester bond between the SUMO-GG motif and the catalytic cysteine residue of the E1-activating enzyme in an ATP-dependent manner; (iii) transfer of the activated SUMO from the E1-activating enzyme to the E2-conjugating enzyme via a thioester linkage between the cysteine residue of the E2 enzyme and the SUMO-GG motif; and (iv) E3 ligase-mediated formation of an isopeptide bond between the carboxyl group of the C-terminal glycine of the SUMO protein and the ε-amino group of the specific lysine residue in the target protein [1,3,4,5]. These SUMOylation steps are schematically illustrated in Figure 1.

The acceptor lysine amino acids in SUMO target proteins are usually located within the consensus motif ΨKxE, with Ψ, K, x and E representing a branched aliphatic amino acid residue, SUMO-conjugating lysine residue, any amino acid residue and glutamic acid residue, respectively [2,3]. Specific SUMO E3 ligases are involved in the SUMOylation of cellular proteins [1,2,3]. SUMO target proteins have been reported in several cell organelles, including the nucleus, endoplasmic reticulum and mitochondria, the cytoplasm and the plasma membrane [2,4]. SUMOylation could affect different aspects of target proteins, including subcellular localization, activity and stability, blocking other lysine-targeting modifications and modulation of protein–protein interaction [1,2,5]. The SUMO modification enzymes and SUMO target proteins, and their effectors, contain a short SUMO interaction motif (SIM) which is pivotal to the relay of SUMOylation consequences [4,6,7]. The SUMO polypeptide also interacts non-covalently with SIM-containing proteins, and regulates their functions [1,3,4,7].

The deSUMOylase (SUMO-cleaving enzyme/isopeptidase) enzymes are pivotal to the maintenance of a cellular pool of readily available free SUMO, as these can release SUMO by cleaving SUMO-substrate bond from SUMOylated proteins, thereby making SUMOylation a dynamic and reversible post-translational modification [1,3,4,8]. Many internal and external cues, including cell cycle stage and thermal and oxidative stress, perturb the levels of cellular SUMOylated proteins (SUMOylome) [2,5,9,10,11,12,13]. The balance of protein SUMOylation in cellular organelles and compartments is maintained by the exquisite regulatory mechanisms, including the differential localization of SUMO-modifying enzymes [1,2,4,5,12]. Although post-translational modifications of proteins, including SUMOylation and ubiquitination, are key players in the complex regulation of cellular processes [7,12,14,15,16], these are not well studied in human fungal pathogens. In this review, our aim is to provide an overview of fungal SUMOylation enzymes and SUMO-target proteins, and their functions in fungal physiology and virulence.

## 2. SUMOylation and Ubiquitination

SUMO belongs to the family of ubiquitin-like proteins which conjugate to and modify cellular proteins, and modulate a wide range of physiological processes [7,14,15]. Sequence-wise, SUMO, a protein of 97 amino acids, is not very similar to ubiquitin, however, it possesses the characteristic ubiquitin-like fold and forms a three-dimensional structure similar to that of ubiquitin [1,7,14]. With regard to the enzymatic steps, protein SUMOylation is quite akin to protein ubiquitination [1,14,15]. Analogous to ubiquitin, SUMO is covalently conjugated to specific lysine residues in target proteins [1,5,14,16]. Furthermore, SUMO also forms poly-SUMOylated chains [1,4,5,17]. Although ubiquitin is mostly associated with protein degradation, SUMOylation does not mark the protein for degradation, but controls the functions of the proteins by modulating other properties, including protein–protein interaction surface alteration [1,2,14,18]. The major similarities and differences between SUMOylation and ubiquitination are listed in Table 1.

Host SUMOylation has been shown to be a key modulator of the pathogen–host interaction, with many bacterial and viral pathogens targeting the host SUMOylation machinery [19,20,21]. Although SUMOylation has been implicated in the regulation of stress responses and the development and differentiation of fungal cells [11,13,22,23], its role in host–fungus interaction and the virulence of medically important fungi is yet to be explored in full. The current review summarizes the key aspects of fungal SUMOylation systems and their role in fungal pathobiology.

## 3. SUMOylation in *Saccharomyces cerevisiae*

SUMOylation is a conserved and essential process in almost all eukaryotes, barring a few organisms including fungi, *Schizosaccharomyces pombe* and *Aspergillus nidulans* [2,22,24]. The SUMOylation process has extensively been studied in the budding yeast *Saccharomyces cerevisiae* [1,5,8,25,26]. Compared to higher eukaryotes, *S. cerevisiae* has a simpler SUMO machinery, represented by a sole SUMO protein (Smt3), two deSUMOylases (Ulp1 and Ulp2), the heterodimeric SUMO-activating enzyme complex consisting of a small non-catalytic subunit Aos1 and a large catalytic subunit Uba2, a sole E2-conjugating enzyme Ubc9 and four E3-SUMO ligases Siz1, Siz2, Cst9 and Mms21 (Table 2) [27,28,29,30,31,32,33,34,35]. Sequence similarity-wise, Smt3 and Ubiquitin proteins in *S. cerevisiae* are 17% identical [28]. Of SUMOylation components, Ubc9 is a key regulator of substrate specificity, as it possesses binding sites for Smt3, E1-activating enzyme, E3 ligases and SUMO target proteins [30,36,37]. SUMO ligases contain the SP-RING domain which plays an important role in binding to Ubc9 directly [38,39]. Furthermore, multiple domains have been implicated in substrate specificity of the Siz1 ligase [40]. Importantly, genes coding for Smt3, Ulp1, Aos1, Uba2, Ubc9 and Mms21 proteins are non-dispensable for cell growth in *S. cerevisiae* [27,28,30,41,42,43]. SUMOylation modulates several cellular processes, including chromosome segregation, DNA replication, cell cycle progression, telomere position effect, and septin ring and nuclear pore dynamics [1,8,26,44]. For a detailed overview of the role of *S. cerevisiae* SUMOylation machinery in fundamental cellular processes, the reader is referred to other reviews [1,5,26,45].

## 4. SUMOylation in Human Pathogenic Fungi

Yeasts and filamentous fungi are emerging as important human pathogens, and can be the fourth most common cause of hospital-acquired bloodstream infections [46,47,48,49]. Fungal infections are associated with a high economic burden worldwide [50,51,52]. The predominant fungal infections are of two types: superficial and invasive [47]. Superficial infections are typified by infections of the skin, hair, nails or the mucosal membrane caused mainly by dermatophytes (species of *Trichophyton*, *Microsporum* and *Epidermophyton*) or pathogenic yeasts (*Candida* species) [47,53]. Contrarily, invasive fungal infections are deep-seated and life-threatening, with a mortality rate of up to 95% [47,54].

The incidence of invasive mycoses caused by opportunistic fungi has increased dramatically in last two decades [54,55,56]. This increase has been attributed to the increase in the number of immunocompromised patients, the use of immunosuppressants, broad-spectrum antibiotics and prophylactic antifungals, and the emergence of drug resistance in pathogenic fungi [54,57,58]. Invasive fungal infections are primarily caused by species of *Candida*, *Aspergillus*, *Pneumocystis* and *Cryptococcus* [47,48,54,56]. Cryptococcal meningitis, caused predominantly by *Cryptococcus neoformans*, and respiratory infections including pneumonia, due to *Pneumocystis jirovecii*, are prevalent in Human Immunodeficiency Virus (HIV)-infected patients [47,59,60]. Furthermore, invasive aspergillosis involving severe infections of the lungs are primarily caused by *A. fumigatus* and associated with a mortality rate of < 90% in undiagnosed or late-diagnosed cases [47,61,62]. *A. flavus*, besides being the second most prevalent causative agent of invasive aspergillosis after *A. fumigatus*, also infects several crops and contributes substantially to aflatoxin-related deaths [63]. Other medically important fungi, causing deep-seated infections of visceral organs, such as the lungs, include *Blastomyces dermatitidis*, *Paracoccidioides brasiliensis*, *Histoplasma capsulatum* [64,65]. The SUMOylation process in these important human fungal pathogens is either uncharacterized or yet to be fully elucidated.

A few recent studies have yielded some insights into the SUMOylation machinery in *C. albicans*, *C. glabrata* and *A. flavus* [11,13,23], however, information on the SUMOylation apparatus in other important human fungal pathogens, including *A. fumigatus*, *Cryptococcus neoformans*, *Cryptococcus gattii,* and *H. capsulatum*, is largely lacking. As a first step towards reviewing fungal SUMOylation systems, we have identified, via BLASTP analysis, orthologs of *S. cerevisiae* proteins that are involved in SUMOylation in four medically relevant fungi (Table 2). The important characteristic features of these proteins, along with known SUMOylation components in *C. albicans*, *C. glabrata* and *A. nidulans*, are described in Table 2.

Of note, all the predicted SUMOylation machinery components in *Cryptococcus neoformans* and *H. capsulatum* have the catalytic residues and domains essential for their enzymatic activity, except for CnAos1, HcAos1 and HcUba2. The HcUba2 lacks the conserved cysteine residue, which has been shown to be essential for SUMO binding in *S. cerevisiae* [27], while CnAos1 and HcAos1 lack the Uba2-interacting RLW (arginine-leucine-tryptophan) motif [66] (Table 2). A chemical–genetic screen has recently implicated the SUMO-activating enzyme CnAos1, in lithium tolerance in *Cryptococcus neoformans*, as a mutant lacking CnAos1 displayed four-fold enhanced growth in the presence of excess lithium chloride [67].

## 5. SUMOylation in *A. nidulans* and *A. flavus*

Among *Aspergillus* spp., SUMOylation machinery components have been identified and studied in the pathogenic species, *A. flavus* and the model species *A. nidulans* [22,62,68,69]. The known SUMOylation components in *A. nidulans* are the sole Smt3 protein (SumO), SumO activating enzymes AosA and UbaB, SumO-specific isopeptidases, UlpA and UlpB, the E2-conjugating enzyme UbcN, and the E3 enzyme SizA [69,70]. The SumO protein in *A. nidulans* is processed by the SUMO protease UlpB, while the UlpA protease is largely involved in the de-SUMOylation process, as the *ulpAΔ* and *ulpBΔ* mutants contained 25-fold higher levels and no SUMO-conjugated proteins, respectively, compared to *wild-type* cells [69,70]. Furthermore, although *sumO* deletion in *A. nidulans* did not affect cell viability, it resulted in growth attenuation, formation of small colonies with ragged edges, sensitivity to DNA damage stress, decreased conidiation, substantially altered secondary metabolite production and self-sterility [22,68,69,70]. The *sumOΔ* mutant also exhibited the derepression of the light-induced sexual development process [69]. Contrary to the *sumOΔ* mutant phenotypes, *sumO* overexpression had no effect on cell growth [22]. In addition, similar to *S. cerevisiae* [71,72], the localization of GFP-SumO was found to be cell cycle-dependent, with distinct SUMO puncta present in the nucleoplasm during interphase and telophase [22].

The deletion of *ulpA* in *A. nidulans* resulted in diminished asexual spore production, and immature cleistothecia formation, despite the increased formation of the sexual fruiting body during asexual development [69,70]. The UlpB protease-encoding gene loss also led to similar asexual and sexual developmental defects, along with highly attenuated growth [69,70]. In addition to UlpA and UlpB, a deneddylase enzyme, DenA, also contains the Ulp domain (includes the core cysteine protease domain), and *denA* deletion resulted in developmental phenotypes similar to the *ulpAΔ* mutant [69,73,74]. Intriguingly, although DenA shows similarity to the SUMO isopeptidase Senp8, it is known to cleave Nedd8, another ubiquitin-like post-translational protein modifier [73,74]. Consistently, despite DenA and UlpA performing similar functions in the multicellular development of *A. nidulans*, DenA could not completely rescue defects arising from the lack of UlpA, indicating that it is not a bonafide SUMO-deconjugase [69]. Moreover, a set of 56 proteins has been found to interact with the TAP-tagged SUMO protein, including many SUMO-modification enzymes [69]. Lastly, AosA and UbaB, have been reported to be dispensable in *A. nidulans,* however, lack of either of these two E1-activating enzymes or the sole E2 enzyme UbcN, resulted in the loss of SUMOylation along with slow growth, impaired conidia production and other developmental defects [69,70]. Intriguingly, deletion of *sizA* and *sizB* either singly or in combination neither had an effect on growth nor on conidiation [69,70]. In contrast, the *mmsUΔ* mutant exhibited slow growth as well as defective conidiation [70]. Of note, the proficiency of *sizΔ* mutants in conidiospore and cleistothecia formation may reflect functional redundancy among SUMO ligases in *A. nidulans* [69,70]. In addition, using the new SUMOlock technique, a set of 149 SUMOylated proteins have recently been identified in *A. nidulans* which are primarily involved in transcription, RNA processing and DNA replication and repair [70], indicating the pivotal role of SUMOylation in the regulation of nucleic acid metabolic processes.

Compared to *A. nidulans*, functional information on the SUMOlyation machinery is limited in *A. flavus*. Intriguingly, the sole SUMO protein in *A. flavus*, AfSumO, is known to possess the characteristic diglycine residue motif, GG, but it lacks the C-terminal stretch of amino acid residues that keep the GG motif hidden, and, thus, may not require processing prior to activation [23]. The lack of C-terminus amino acid residues has also been reported in the hypothetical SUMO proteins of other *Aspergillus* species, including *A. fumigatus* [23], however, the role that predicted SUMO-processing proteases play in these fungi remains to be determined.

Furthermore, SUMOylation in *A. flavus* has been reported to be temperature-dependent, as increased amounts of SUMO-conjugated proteins were observed in mycelia upon growth at 37 °C, compared to those at 29 °C [23]. However, *AfsumO* loss had no effect on cell growth at either temperature, but it made *A. flavus* cells more sensitive to DNA damage and oxidative stress [23]. *AfsumO* deletion also led to a lower rate of conidiation and decreased production of secondary metabolites, aflatoxins AFB1 and AFB2 [23]. Contrarily, the *AfsumO*-overexpressing strain grew slightly better under stress conditions, formed more conidia and produced two-fold higher levels of aflatoxins [23]. The effect of SUMOylation on aflatoxin production was attributed to the differential expression of the genes encoding transcriptional regulators and enzymes involved in aflatoxin biosynthesis [23]. Of note, the role of SUMOylation in sclerotia formation also appears to be modulated by temperature, with the *AfsumOΔ* mutant (lacks the SUMO protein) displaying increased and decreased sclerotia production at 29 °C and 37 °C, respectively [23]. Lastly, mCherry-tagged AfSumO protein, along with its target proteins, were found both in the cytoplasm and the nucleus [23].

## 6. SUMOylation in *Candida albicans* and *Candida glabrata*

*Candida* bloodstream infections (BSIs), a frequent occurrence in immunocompromised individuals, are associated with an average mortality rate of about 40% [47,54,56,75]. The incidence of opportunistic candidemia has increased substantially worldwide in the last two decades, with *Candida albicans* being the most dominant species followed by the *non-albicans* species, represented largely by *C. glabrata*, *C. tropicalis* and *C. parapsilosis*, and rapidly emerging *C. auris* [47,54,75,76]. *C. glabrata* accounts for 10%–35% of *Candida* bloodstream infections, based on the geographical distribution [75,76,77,78,79]. *C. albicans* is a diploid organism, with key virulence traits of activity of secreted proteases, mating, morphological and colony switching and biofilm formation [80,81,82]. Contrarily, *C. glabrata* is haploid in nature, and phylogenetically more closely related to *S. cerevisiae* than to *C. albicans* [83,84]. Intriguingly, *C. glabrata* neither secretes aspartyl proteases nor switches between yeast and hyphal forms, the two major attributes that allow fungal pathogens to establish successful infections [81,82,85,86]. The major virulence factors of *C. glabrata* include multigene families encoding at least seventeen cell surface epithelial adhesins (EPA) and eleven glycosylphosphatidylinositol (GPI)-linked aspartyl proteases [85,86,87]. Additionally, *C. glabrata* possesses a unique ability to survive high levels of diverse stresses and proliferate in host macrophages without causing any harm to macrophage cells [86,88,89]. Despite these differences, *C. glabrata* and *C. albicans* share many virulence traits, including biofilm formation, metabolic plasticity and colony switching [81,82,85,86]. The post-translational modifications, including phosphorylation and glycosylation, have been implicated in the virulence of human fungal pathogens, including *Candida* spp. [90]. Over the past decade, SUMOylation has been studied in two *Candida* species, *C. albicans* and *C. glabrata*, and its role in *Candida* pathogenesis is beginning to be appreciated [11,13,91,92].

As shown in Table 2, *C. albicans* has a sole Smt3 SUMO protein, three Ulp domain-containing proteins CaUlp1, CaUlp2 and CaUlp3, a heterodimeric E1 activating enzyme complex of CaAos1 and CaUba2, the E2 enzyme CaUbc9, and E3 ligases CaSiz1, CaMms21, CaCst9 and CaWos1. Among these SUMOylation components, CaSmt3, CaAos1, CaUba2, CaUbc9 and CaMms21 are not essential for cell viability [92].

Of the SUMO proteases, all three CaUlp1, CaUlp2 and CaUlp3, when expressed in *Pichia pastoris*, displayed SUMO-processing activity [93]. Moreover, while both RNA and protein levels of CaUlp1 and CaUlp3 proteases were observed in the yeast and hyphal cells of *C. albicans*, CaUlp2 transcript or protein expression was detectable in neither morphological form [93]. These data suggest that CaUlp1 and CaUlp3 may be the major proteases for CaSmt3 under normal growth conditions. In addition, the E2-conjugating enzyme CaUbc9 has been shown to physically interact with the SUMO E3 ligase CaWos1 [91].

Through phenotypic and molecular analysis, SUMOylation has been implicated in the regulation of many processes including filamentation, with yeast–hyphae morphogenesis being essential for virulence in *C. albicans* [11,81,94]. CaSmt3 and CaAos1 have been demonstrated to act as repressors of hyphae formation in *C. albicans* [94]. Additionally, the *C. albicans smt3Δ/smt3Δ* cells exhibited defects in cell separation and nuclear segregation, and formed elongated buds [11]. The *smt3Δ/smt3Δ* cells were also defective in their formation of hyphae in response to serum, and activation of the Protein Kinase C-mediated cell wall integrity pathway in response to stresses [11]. In agreement with this, the *smt3Δ/smt3Δ* cells were found to be sensitive to several stresses including thermal, oxidative, unfolded protein, cell wall and antifungal stresses, and contained high chitin in the cell wall [11]. Importantly, the *C. albicans mms21Δ/mms21Δ* mutant also exhibited slow growth, thermal stress susceptibility, nuclear segregation defects, increased invasiveness, unregulated filamentation and diminished recovery from DNA damage [92]. This mutant also showed sensitivity to cell wall stressors, and azole and echinocandin antifungal drugs [92].

*C. glabrata* possesses orthologs of all *S. cerevisiae* SUMOylation components [13]. The *C. glabrata* CgSmt3, CgUba2, CgAos1, CgUbc9, CgSiz1, CgSiz2, CgMms21, CgCst9, CgUlp1 and CgUlp2 proteins showed sequence identities of 81%, 62%, 55%, 89%, 42%, 34%, 37%, 49%, 52% and 43% with their respective *S. cerevisiae* SUMO counterparts, respectively (Table 2). Overall, the *C. glabrata* SUMO machinery is quite similar to the *S. cerevisiae* SUMO system, with one exception being the lack of a SAP [Scaffold attachment factor (SAF)-A/B-Acinus-Protein inhibitor of activated STAT (PIAS)] domain in the CgSiz1 enzyme. As the SAP domain is involved in the nuclear retention of the *S. cerevisiae* Siz1 ligase [13], its absence in CgSiz1 may hint towards non-nuclear substrates of the CgSiz1 enzyme. The *CgSMT3* gene was found to be essential for cell growth of *C. glabrata* [13].

Furthermore, functional conservation between *C. glabrata* and *S. cerevisiae* SUMOylation machinery has also been reported, as CgSmt3 and CgUlp2 could restore the cell viability and growth defects of *Scsmt3Δ* and *Sculp2Δ* mutants, respectively [13]. The *Cgulp2Δ* mutant showed slow growth, sensitivity to multiple stresses, including thermal, DNA damage and oxidative stress, elevated chitin levels, diminished adherence to host epithelial cells, reduced replication in macrophages and poor colonization in a murine systemic candidiasis model, indicating a pivotal role for CgUlp2 in pathogenesis of *C. glabrata* [13]. In contrast, the *Cgsiz1Δ* mutant had no discernible phenotype while the *Cgsiz2Δ* and *Cgsiz1Δsiz2Δ* mutants displayed sensitivity to DNA damage caused by UV radiation and MMS (methyl methanesulfonate), implicating CgSiz2 in the survival of DNA damage stress [13]. Surprisingly, despite the antagonistic functions of CgUlp2 and CgSiz1-Siz2 enzymes, both *Cgsiz1Δsiz2Δ* and *Cgulp2Δ* mutants lacked any detectable SUMOylated proteins [13]. The *Cgulp2Δ* mutant also had no free SUMO protein [13]. Although the molecular basis for this paradoxical result is yet to be elucidated, these data highlight the complex regulation of the cellular SUMOylation system. Lastly, the inability to generate strains deleted for *CgAOS1*, *CgUBA2*, *CgUBA9*, *CgMMS21* and *CgULP1* genes could reflect their essentiality for the cell viability of *C. glabrata* [13].

In terms of the nature and localization of SUMO-target proteins, SUMO modification of septins has not been observed in *C. albicans*, unlike *S. cerevisae* [29,71,95]. However, CaSmt3 has been reported to localize at bud necks in the yeast form, and at septation sites in the mature hyphae, indicating the SUMO-conjugation of other bud neck and/or septin-associated proteins [95]. Similarly, SUMO ligases CgSiz1 and CgSiz2, and SUMO proteases CgUlp1 and CgUlp2, in *C. glabrata* displayed predominantly nuclear localization, while the CgSmt3 protein was found to be uniformly distributed throughout the cell [13].

**Figure 2:**
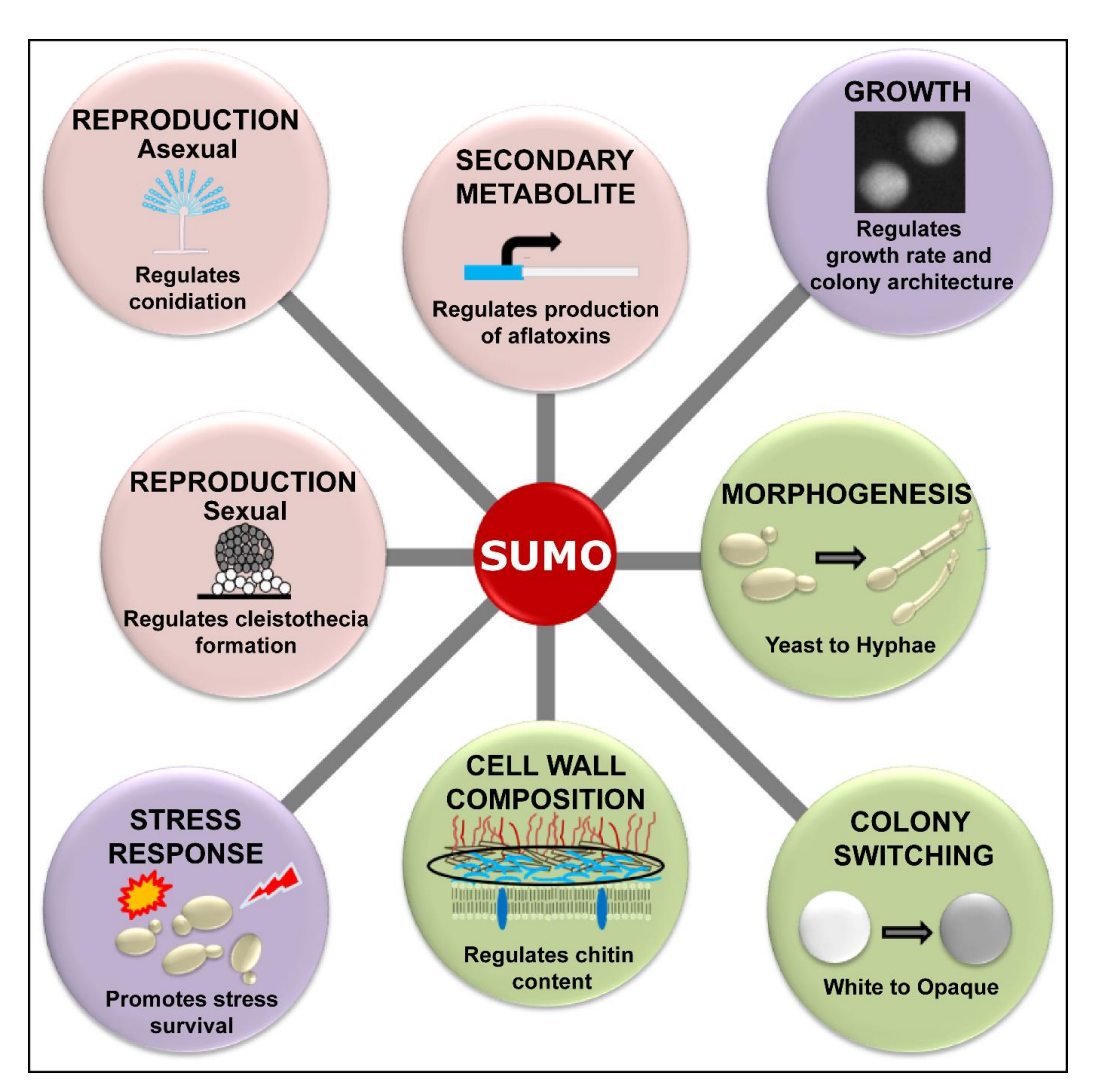
Schematic representation of diverse physiological processes, that are known to be regulated by SUMOylation in the human pathogenic fungi. SUMOylation regulates growth profiles and survival of different stresses in species of both *Aspergillus* and *Candida*. Additionally, while SUMOylation modulates sexual and asexual reproduction, and secondary metabolite production in *Aspergillus*, it regulates colony and morphology switching, and maintenance of cell wall composition in *Candida*.

**Figure 3:**
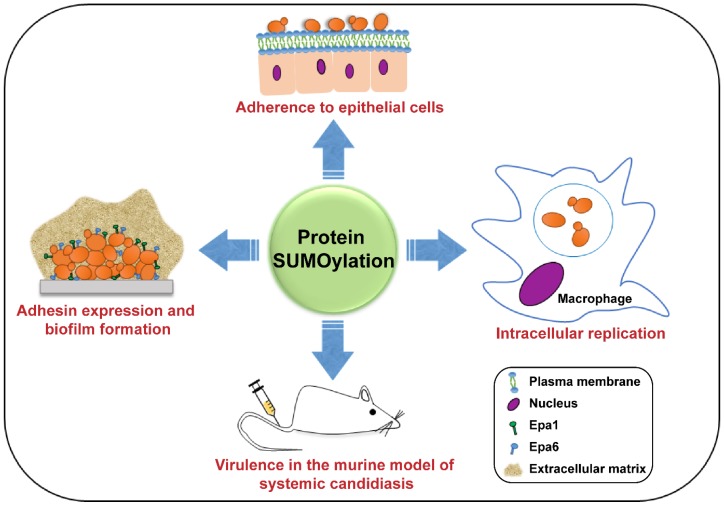
A schematic illustrating the roles of SUMOylation in the pathogenesis of *C. albicans* and *C. glabrata*. SUMOylation is required for adherence to epithelial cells, biofilm formation and intracellular proliferation of *C. glabrata* in human macrophages. SUMOylation also modulates the virulence of *C. albicans* and *C. glabrata* in mice.

To summarize, the process of SUMOylation is important for cell division, growth and stress response in human pathogenic fungi studied so far [11,13,23]. However, the SUMO-encoding *SMT3* gene does not appear to be essential in all fungi, as *SMT3* is required for viability in *S. cerevisiae* [28] and *C. glabrata* [13], but not in *C. albicans* [11] and *A. nidulans* [22]. SUMO enzymes are also required for survival of many stresses, the activation of the cell wall integrity MAPK (mitogen-activated protein kinase) pathway and the negative regulation of the cell wall chitin in *C. glabrata* and *C. albicans* [11,13]. The known roles of SUMOylation in fungal cell physiology and virulence are depicted in Figure 2 and Figure 3, respectively.

## 7. SUMOylated Target Proteins

The work by Leach *et al.* has shed light on potential SUMO-target proteins in *C. albicans* [11]. Using an N-terminally FLAG-tagged SUMO, Leach *et al.* found 31 proteins to be SUMOylated through a proteomic screen [11]. These proteins were involved in cellular stress response, cytoskeleton organization, secretion, metabolism and endocytosis [11]. Two of the identified SUMOylation targets were heat shock proteins Hsp60 and Hsp104, and mutations of the consensus SUMOylation residue lysine in Hsp60 and Hsp104 proteins mirrored the morphology defect and thermal stress sensitivity, respectively, of the *smt3Δ/smt3Δ* mutant, underscoring the role of SUMOylation in the cellular functions of Hsp60 and Hsp104 [11]. Moreover, SUMOylation of the major transcriptional factor of white–opaque phenotypic switching, CaWor1, is regulated by the SUMO E3 ligase CaWos1 (Wor1 SUMO ligase 1), and the loss of CaWor1 SUMOylation led to impaired white to opaque switching and a less stable opaque phase phenotype [91]. CaWos1 was also implicated in the cellular carbon dioxide (CO_2_)-sensing response, as elevated CO_2_ concentration led to the upregulation of the *CaWOS1* gene in a Flo8-dependent manner, and deletion of *CaWOS1* caused significant decrease in the white to opaque switching frequency under high CO_2_ conditions [91]. Contrary to the *wos1Δ/wos1Δ* mutant, the colony morphology of the *smt3Δ/smt3Δ* mutant was heterogeneous, consisting of equal numbers of opaque, white and wrinkled colonies, with a higher switching rate among different cell states [11,91]. Of note, *CaWOS1* loss had no effect on the virulence of *C. albicans* in mice, however, its overexpression led to attenuated virulence [91]. Consistent with the central role of SUMOylation in modulation of the virulence traits of *C. albicans*, a potential SUMOylation site in the yeast phase-specific protein CaSlp3 (Stomatin Like Protein 3), that may be involved in its targeting to the plasma membrane and the vacuole, has recently been identified [96]. Of note, Slp3 in *C. albicans* has also been shown to be an oxidative stress response protein, whose overproduction resulted in mitochondrial depolarization and apoptotic-like cell death upon prolonged oxidative stress [96]. Figure 3 schematically represents the roles of SUMOylation in the virulence of *Candida* spp.

## 8. SUMOylation and Stress Response

SUMOylation is a dynamic post-translational protein modification, with cells responding to stressful conditions by altering their SUMOylome. The levels of SUMOlyated proteins were found to be significantly elevated in *C. glabrata* cells exposed to ethanol stress, DNA damaging agents and the macrophage internal milieu [13]. Similarly, heat shock, oxidative and cell wall stress and hyphae-inducing conditions altered the SUMOylome in *C. albicans* [11]. These preliminary studies point towards a regulatory role for SUMOylation in sensing and/or relaying cellular stress signals in pathogenic fungi, which may aid cells mount an appropriate response to survive stressful environmental conditions.

## 9. Future Perspectives

Investigating the role of post-translational modifications, including SUMOylation, in fungal virulence is a rapidly growing field. The recent advancement in protein identification technologies has promoted the use of high-throughput proteomic screens to analyze the virulence traits of human fungal pathogens. These mass spectrometry-based techniques are likely to be beneficial in the identification of the dynamic SUMOylome, as well as the key regulators of cellular SUMOlyation networks in pathogenic fungi. Two crucial areas, that are yet to be explored, are the contribution of the environmental cue-specific rapid subcellular distribution of the SUMO-modification enzymes to the rewiring of cellular signaling circuits, and the possibility of SUMO-modification enzymes as antifungal drug targets. A better understanding of the underlying molecular and biochemical mechanisms by which protein SUMOylation aids the pathogenic fungi in adapting to diverse stresses, acquiring drug resistance, maintaining genomic integrity and expressing virulence factors may lead to better intervention strategies for the diagnosis and control of fungal infections.

## Figures and Tables

**Table 1:** A comparison of SUMOylation and ubiquitination ^#^.

	Characteristics	SUMOylation	Ubiquitination
Similarities	Attachment site	Lysine	Lysine
	Modifier maturation	Required	Required
	Enzymes involved	E1, E2 and E3	E1, E2 and E3
	Reversibility	Yes	Yes
	Energy consumption	Yes	Yes
Differences	Modifier size	~11 kDa	~8.6 kDa
	Isoform	Multiple	No
	Consensus motif	Ψ-K-x-E	No consensus
	E1-activating enzyme	Heterodimeric	Monomeric
	Number of E2-conjugating enzymes	Single	Multiple
	Number of E3-ligases	Few (1–4)	Hundreds
	E3-ligase requirement for the conjugation reaction	Not essential	Essential
	Lysine residue of the modifier protein involved in poly-chain formation	K11	K6, K11, K27, K29, K33, K48, K63

^#^ This table is prepared with the information gathered from articles [1,2,3,15,16,17].

**Table 2:** A list of SUMOylation components in seven fungi ^#^.

Fungal Pathogens	Systematic ORF	Size (kDa)	Catalytic Motif	Description	% Identitywith with *S. cerevisiae* Ortholog	Common/Systematic Name in *S. cerevisiae*
**Small Ubiquitin-Like Modifier (SUMO)**						
*C. albicans*	*C1_11330C_A*	11.1	Present	Verified	61.39	*SMT3/* *YDR510W*
*C. glabrata*	*CAGL0K05731g*	12.2	Present	Uncharacterized	81.31	
*A. nidulans*	*AN1191*	10.4	Present	Verified	53.75	
*C. neoformans*	*CNC00390*	11.3	Present	Uncharacterized	46.74	
*H. capsulatum*	*HCAG_01770*	10.8	Present	Uncharacterized	53.85	
*B. dermatitidis*	*BDDG_01171*	10.6	Present	Uncharacterized	55.70	
*P. brasiliensis*	*PABG_00491*	10.6	Present	Uncharacterized	52.75	
**SUMO-Activating Enzyme (E1)**						
*C. albicans*	*C1_08020W_A*	70.9	Present	Uncharacterized	43.75	*UBA2*/ *YDR390C*
*C. glabrata*	*CAGL0M01606g*	71.1	Present	Uncharacterized	62.09	
*A. nidulans*	*AN2450*	67.7	Present	Verified	42.08	
*C. neoformans*	*CNF00770*	72.7	Present	Uncharacterized	37.88	
*H. capsulatum*	*HCAG_04925*	65.1	Absent	Uncharacterized	35.36	
*B. dermatitidis*	*BDDG_04072*	68.7	Present	Uncharacterized	38.47	
*P. brasiliensis*	*PABG_04604*	69.6	Present	Uncharacterized	44.49	
*C. albicans*	*CR_02770C_A*	43.3	Present	Uncharacterized	35.05	*AOS1*/ *YPR180W*
*C. glabrata*	*CAGL0G09889g*	37.8	Present	Uncharacterized	54.94	
*A. nidulans*	*AN2298*	42.2	Present	Verified	32.75	
*C. neoformans*	*CNN00720*	37.7	Absent	Uncharacterized	39.04	
*H. capsulatum*	*HCAG_08393*	38.5	Absent	Uncharacterized	32.14	
*B. dermatitidis*	*BDDG_02776*	40.0	Present	Uncharacterized	34.19	
*P. brasiliensis*	*PABG_06750*	40.1	Present	Uncharacterized	36.21	
**SUMO-Conjugating Enzyme (E2)**						
*C. albicans*	*CR_08560C_A*	25.7	Present	Verified	69.03	*UBC9*/ *YDL064W*
*C. glabrata*	*CAGL0D00814g*	18.0	Present	Uncharacterized	88.54	
*A. nidulans*	*AN4399*	18.0	Present	Verified	63.01	
*C. neoformans*	*CNI02210*	18.2	Present	Uncharacterized	57.90	
*H. capsulatum*	*HCAG_05621*	17.9	Present	Uncharacterized	62.33	
*B. dermatitidis*	*BDDG_09778*	18.0	Present	Uncharacterized	63.01	
*P. brasiliensis*	*PABG_04136*	18.0	Present	Uncharacterized	57.79	
**SUMO Ligases (E3)**						
*C. albicans*	*C1_01560W_A*	174.5	Present	Uncharacterized	27.87	*SIZ1*/ *YDR409W*
*C. glabrata*	*CAGL0F02783g*	94.5	Present	Uncharacterized	41.64	
*A. nidulans*	*AN10822*	55.9	Present	Verified	32.23	
*C. neoformans*	*CNM02250*	88.1	Present	Uncharacterized	28.25	
*H. capsulatum*	*HCAG_06903*	52.2	Present	Uncharacterized	33.98	
*B. dermatitidis*	*BDDG_09007*	59.0	Present	Uncharacterized	31.10	
*P. brasiliensis*	*PABG_05394*	58.9	Present	Uncharacterized	30.20	
*C. albicans*	Absent	-	-	-	-	*SIZ2*/ *YOR156C*
*C. glabrata*	*CAGL0L04290g*	83.2	Present	Uncharacterized	33.72	
*A. nidulans*	*AN4497*	123.5	Present	Uncharacterized	26.44	
*C. neoformans*	Absent	-	-	-	-	
*H. capsulatum*	Absent	-	-	-	-	
*B. dermatitidis*	Absent	-	-	-	-	
*P. brasiliensis*	Absent	-	-	-	-	
*C. albicans*	*C3_06200C_A*	31.3	Present	Uncharacterized	31.67	*MMS21*/ *YEL019C*
*C. glabrata*	*CAGL0M03267g*	30.8	Present	Uncharacterized	37.04	
*A. nidulans*	*AN10240*	56.1	Present	Uncharacterized	33.01	
*C. neoformans*	*CND02680*	37.0	Present	Uncharacterized	24.47	
*H. capsulatum*	*HCAG_05688*	55.5	Present	Uncharacterized	31.82	
*B. dermatitidis*	*BDDG_05774*	54.6	Present	Uncharacterized	33.75	
*P. brasiliensis*	Absent	-	-	-	-	
*C. albicans*	*C2_05900W_A*	41.8	Present	Uncharacterized	33.77	*CST9*/ *YLR394W*
*C. glabrata*	*CAGL0C02629g*	40.1	Present	Uncharacterized	48.77	
*A. nidulans*	Absent	-	-	-	-	
*C. neoformans*	Absent	-	-	-	-	
*H. capsulatum*	*HCAG_01117*	24.2	Absent	Uncharacterized	37.50	
*B. dermatitidis*	Absent	-	-	-	-	
*P. brasiliensis*	Absent	-	-	-	-	
*C. albicans*	*C4_04420W_A*	57.1	Present	Verified	100.00	WOS1 *
*C. glabrata*	Absent	-	-	-	-	
*A. nidulans*	Absent	-	-	-	-	
*C. neoformans*	Absent	-	-	-	-	
*H. capsulatum*	*HCAG_04523*	112.8	Present	Uncharacterized	33.33	
*B. dermatitidis*	*BDDG_13222*	68.2	Present	Uncharacterized	32.56	
*P. brasiliensis*	*PABG_01044*	123.4	Present	Uncharacterized	30.19	
**SUMO Proteases**						
*C. albicans*	*C3_03550C_A*	40.5	Present	Verified	38.43	*ULP1*/ *YPL020C*
*C. glabrata*	*CAGL0L08646g*	68.2	Present	Uncharacterized	51.89	
*A. nidulans*	*AN2689*	107.3	Present	Verified	28.29	
*C. neoformans*	*CNL03980*	55.5	Present	Uncharacterized	30.33	
*H. capsulatum*	*HCAG_06354*	28.6	Present	Uncharacterized	24.28	
*B. dermatitidis*	*BDDG_05156*	114.3	Present	Uncharacterized	29.19	
*P. brasiliensis*	*PABG_00907*	124.1	Present	Uncharacterized	27.76	
*C. albicans*	*C3_00280C_A*	101.3	Present	Verified	37.41	*ULP2*/ *YIL031W*^¶^
*C. glabrata*	*CAGL0J02464g*	104.1	Present	Uncharacterized	44.88	
*A. nidulans*	*AN8192*	125.9	Present	Verified	34.02	
*C. neoformans*	*CND00680*	170.0	Present	Uncharacterized	28.13	
*H. capsulatum*	*HCAG_00522*	138.8	Present	Uncharacterized	28.71	
*B. dermatitidis*	*BDDG_05054*	139.4	Present	Uncharacterized	26.99	
*P. brasiliensis*	*PABG_04092*	137.2	Present	Uncharacterized	26.67	

^#^ The orthologs of S. cerevisiae SUMO protein and SUMOylation enzmyes were identified, via BLASTP analysis, in Candida albicans, Candida glabrata, Aspergillus nidulans, Cryptococcus neoformans, Histoplasma capsulatum, Blastomyces dermatitidis and Paracoccidioides brasiliensis. The features of identified proteins including the presence of the conserved catalytic motif were extracted from the Candida Genome Database (CGD), Aspergillus Genome Database (AGD), and UniProt Database. * Due to the absence of Wos1 in S. cerevisiae, the sequence of C. albicans Wos1 was used for BLASTP analysis. ^¶^ C. albicans possesses an additional SUMO protease, CaUlp3, that is encoded by the CR_03820C_A ORF, and shows homology to Ulp2 of S. cerevisiae.

## References

[1] Johnson E.S. (2004). Protein modification by SUMO. Annu. Rev. Biochem..

[2] Flotho A., Melchior F. (2013). Sumoylation: A regulatory protein modification in health and disease. Annu. Rev. Biochem..

[3] Pichler A., Fatouros C., Lee H., Eisenhardt N. (2017). SUMO conjugation—A mechanistic view. Biomol. Concepts.

[4] Geiss-Friedlander R., Melchior F. (2007). Concepts in sumoylation: A decade on. Nat. Rev. Mol. Cell Biol..

[5] Hay R.T. (2005). SUMO: A history of modification. Mol. Cell.

[6] Song J., Durrin L.K., Wilkinson T.A., Krontiris T.G., Chen Y. (2004). Identification of a SUMO-binding motif that recognizes SUMO-modified proteins. Proc. Natl. Acad. Sci..

[7] Kerscher O. (2007). SUMO junction—What’s your function?. EMBO Rep..

[8] Palancade B., Doye V. (2008). Sumoylating and desumoylating enzymes at nuclear pores: Underpinning their unexpected duties?. Trends Cell Biol..

[9] Zhou W., Ryan J.J., Zhou H. (2004). Global analyses of sumoylated proteins in *Saccharomyces cerevisiae*. Induction of protein sumoylation by cellular stresses. J. Biol. Chem..

[10] Golebiowski F., Matic I., Tatham M.H., Cole C., Yin Y., Nakamura A., Cox J., Barton G.J., Mann M., Hay R.T. (2009). System-wide changes to SUMO modifications in response to heat shock. Sci. Signal..

[11] Leach M.D., Stead D.A., Argo E., Brown A.J.P. (2011). Identification of sumoylation targets, combined with inactivation of SMT3, reveals the impact of sumoylation upon growth, morphology, and stress resistance in the pathogen *Candida albicans*. Mol. Biol. Cell.

[12] Guo C., Henley J.M. (2014). Wrestling with stress: Roles of protein SUMOylation and deSUMOylation in cell stress response. IUBMB Life.

[13] Gujjula R., Veeraiah S., Kumar K., Thakur S.S., Mishra K., Kaur R. (2016). Identification of components of the SUMOylation machinery in *Candida glabrata*. J. Biol. Chem..

[14] Wilson V.G., Heaton P.R. (2008). Ubiquitin proteolytic system: Focus on SUMO. Expert Rev. Proteomics.

[15] Pickart C.M., Eddins M.J. (2004). Ubiquitin: Structures, functions, mechanisms. Biochim. Biophys. Acta.

[16] Ikeda F., Dikic I. (2008). Atypical ubiquitin chains: New molecular signals. “Protein Modifications: Beyond the Usual Suspects” Review Series. EMBO Rep..

[17] Tatham M.H., Jaffray E., Vaughan O.A., Desterro J.M.P., Botting C.H., Naismith J.H., Hay R.T. (2001). Polymeric chains of SUMO-2 and SUMO-3 are conjugated to protein substrates by SAE1/SAE2 and Ubc9. J. Biol. Chem..

[18] Nie M., Boddy M.N. (2016). Cooperativity of the SUMO and Ubiquitin pathways in genome stability. Biomolecules.

[19] Citro S., Chiocca S. (2010). *Listeria monocytogenes*: A bacterial pathogen to hit on the SUMO pathway. Cell Res..

[20] Wimmer P., Schreiner S., Dobner T. (2012). Human pathogens and the host cell SUMOylation system. J. Virol..

[21] Srikanth C.V., Verma S. (2017). Sumoylation as an integral mechanism in bacterial infection and disease progression. Adv. Exp. Med. Biol..

[22] Wong K.H., Todd R.B., Oakley B.R., Oakley C.E., Hynes M.J., Davis M.A. (2008). Sumoylation in *Aspergillus nidulans*: SumO inactivation, overexpression and live-cell imaging. Fungal Genet. Biol..

[23] Nie X., Yu S., Qiu M., Wang X., Wang Y., Bai Y., Zhang F., Wang S. (2016). *Aspergillus flavus* SUMO contributes to fungal virulence and toxin attributes. J. Agric. Food Chem..

[24] Tanaka K., Nishide J., Okazaki K., Kato H., Niwa O., Nakagawa T., Matsuda H., Kawamukai M., Murakami Y. (1999). Characterization of a fission yeast SUMO-1 homologue, Pmt3p, required for multiple nuclear events, including the control of telomere length and chromosome segregation. Mol. Cell. Biol..

[25] Hannich J.T., Lewis A., Kroetz M.B., Li S.J., Heide H., Emili A., Hochstrasser M. (2005). Defining the SUMO-modified proteome by multiple approaches in *Saccharomyces cerevisiae*. J. Biol. Chem..

[26] Jalal D., Chalissery J., Hassan A.H. (2017). Genome maintenance in *Saccharomyces cerevisiae*: The role of SUMO and SUMO-targeted ubiquitin ligases. Nucleic Acids Res..

[27] Dohmen R.J., Stappen R., McGrath J.P., Forrova H., Kolarov J., Goffeau A., Varshavsky A. (1995). An essential yeast gene encoding a homolog of ubiquitin-activating enzyme. J. Biol. Chem..

[28] Johnson E.S., Schwienhorst I., Dohmen R.J., Blobel G. (1997). The ubiquitin-like protein Smt3p is activated for conjugation to other proteins by an Aos1p/Uba2p heterodimer. EMBO J..

[29] Johnson E.S., Gupta A.A. (2001). An E3-like factor that promotes SUMO conjugation to the yeast septins. Cell.

[30] Johnson E.S., Blobel G. (1997). Ubc9p is the conjugating enzyme for the ubiquitin-like protein Smt3p. J. Biol. Chem..

[31] Li S.J., Hochstrasser M. (1999). A new protease required for cell-cycle progression in yeast. Nature.

[32] Li S.-J., Hochstrasser M. (2000). The yeast ULP2 (SMT4) gene encodes a novel protease specific for the ubiquitin-like Smt3 protein. Mol. Cell. Biol..

[33] Bylebyl G.R., Belichenko I., Johnson E.S. (2003). The SUMO isopeptidase Ulp2 prevents accumulation of SUMO chains in yeast. J. Biol. Chem..

[34] Cheng C.H., Lo Y.H., Liang S.S., Ti S.C., Lin F.M., Yeh C.H., Huang H.Y., Wang T.F. (2006). SUMO modifications control assembly of synaptonemal complex and polycomplex in meiosis of *Saccharomyces cerevisiae*. Genes Dev..

[35] Hoch N.C., Santos R.S., Rosa R.M., Machado R.M., Saffi J., Brendel M., Henriques J.A.P. (2008). Allelism of *Saccharomyces cerevisiae* gene PSO10, involved in error-prone repair of psoralen-induced DNA damage, with SUMO ligase-encoding MMS21. Curr. Genet..

[36] Bencsath K.P., Podgorski M.S., Pagala V.R., Slaughter C.A., Schulman B.A. (2002). Identification of a multifunctional binding site on Ubc9p required for Smt3p conjugation. J. Biol. Chem..

[37] Van Waardenburg R.C.A.M., Duda D.M., Lancaster C.S., Schulman B.A., Bjornsti M.-A. (2006). Distinct functional domains of Ubc9 dictate cell survival and resistance to genotoxic stress. Mol. Cell. Biol..

[38] Takahashi Y., Kahyo T., Toh-e A., Yasuda H., Kikuchi Y. (2001). Yeast Ull1/Siz1 is a novel SUMO1/Smt3 ligase for septin components and functions as an adaptor between conjugating enzyme and substrates. J. Biol. Chem..

[39] Hochstrasser M. (2001). SP-RING for SUMO: New functions bloom for a ubiquitin-like protein. Cell.

[40] Reindle A., Belichenko I., Bylebyl G.R., Chen X.L., Gandhi N., Johnson E.S. (2006). Multiple domains in Siz SUMO ligases contribute to substrate selectivity. J. Cell Sci..

[41] Seufert W., Futcher B., Jentsch S. (1995). Role of a ubiquitin-conjugating enzyme in degradation of S- and M-phase cyclins. Nature.

[42] Li S.J., Hochstrasser M. (2003). The Ulp1 SUMO isopeptidase: Distinct domains required for viability, nuclear envelope localization, and substrate specificity. J. Cell Biol..

[43] Zhao X., Blobel G. (2005). A SUMO ligase is part of a nuclear multiprotein complex that affects DNA repair and chromosomal organization. Proc. Natl. Acad. Sci..

[44] Wohlschlegel J.A., Johnson E.S., Reed S.I., Yates J.R. (2004). Global analysis of protein sumoylation in *Saccharomyces cerevisiae*. J. Biol. Chem..

[45] Dasso M. (2008). Emerging roles of the SUMO pathway in mitosis. Cell Div..

[46] Wisplinghoff H., Bischoff T., Tallent S.M., Seifert H., Wenzel R.P., Edmond M.B. (2004). Nosocomial bloodstream infections in US hospitals: Analysis of 24, 179 cases from a prospective nationwide surveillance study. Clin. Infect. Dis..

[47] Brown G.D., Denning D.W., Gow N.A.R., Levitz S.M., Netea M.G., White T.C. (2012). Hidden killers: Human fungal infections. Sci. Transl. Med..

[48] Limper A.H., Adenis A., Le T., Harrison T.S. (2017). Fungal infections in HIV/AIDS. Lancet Infect. Dis..

[49] Casadevall A. (2018). Fungal diseases in the 21st century: The near and far horizons. Pathog. Immun..

[50] Drgona L., Khachatryan A., Stephens J., Charbonneau C., Kantecki M., Haider S., Barnes R. (2014). Clinical and economic burden of invasive fungal diseases in Europe: Focus on pre-emptive and empirical treatment of *Aspergillus* and *Candida* species. Eur. J. Clin. Microbiol. Infect. Dis..

[51] Mansour Ceesay M., Sadique Z., Harris R., Ehrlich A., Adams E.J., Pagliuca A. (2014). Prospective evaluation of the cost of diagnosis and treatment of invasive fungal disease in a cohort of adult haematology patients in the UK. J. Antimicrob. Chemother..

[52] Benedict K., Jackson B.R., Chiller T., Beer K.D. (2019). Estimation of direct healthcare costs of fungal diseases in the United States. Clin. Infect. Dis..

[53] Woodfolk J.A. (2005). Allergy and dermatophytes. Clin. Microbiol. Rev..

[54] Pfaller M.A., Pappas P.G., Wingard J.R. (2006). Invasive fungal pathogens: Current epidemiological trends. Clin. Infect. Dis..

[55] Sipsas N.V., Kontoyiannis D.P. (2012). Invasive fungal infections in patients with cancer in the intensive care unit. Int. J. Antimicrob. Agents.

[56] Bongomin F., Gago S., Oladele R.O., Denning D.W. (2017). Global and multi-national prevalence of fungal diseases-estimate precision. J. fungi.

[57] Köhler J.R., Hube B., Puccia R., Casadevall A., Perfect J.R. (2017). Fungi that infect humans. Fungal Kingd..

[58] Perlin D.S., Rautemaa-Richardson R., Alastruey-Izquierdo A. (2017). The global problem of antifungal resistance: Prevalence, mechanisms, and management. Lancet Infect. Dis..

[59] Rajasingham R., Smith R.M., Park B.J., Jarvis J.N., Govender N.P., Chiller T.M., Denning D.W., Loyse A., Boulware D.R. (2017). Global burden of disease of HIV-associated *Cryptococcal meningitis*: An updated analysis. Lancet Infect. Dis..

[60] Fisk D.T., Meshnick S., Kazanjian P.H. (2003). *Pneumocystis carinii* pneumonia in patients in the developing world who have acquired immunodeficiency syndrome. Clin. Infect. Dis..

[61] Von Eiff M., Roos N., Schulten R., Hesse M., Zühlsdorf M., van de Loo J. (1995). Pulmonary aspergillosis: Early diagnosis improves survival. Respiration.

[62] Dagenais T.R.T., Keller N.P. (2009). Pathogenesis of *Aspergillus fumigatus* in invasive aspergillosis. Clin. Microbiol. Rev..

[63] Rudramurthy S.M., Paul R.A., Chakrabarti A., Mouton J.W., Meis J.F. (2019). Invasive aspergillosis by *Aspergillus flavus*: Epidemiology, diagnosis, antifungal resistance, and management. J. Fungi.

[64] Castillo C.G., Kauffman C.A., Miceli M.H. (2016). Blastomycosis. Infect. Dis. Clin. North. Am..

[65] De Macedo P.M., De Melo Teixeira M., Barker B.M., Zancopé-Oliveira R.M., Almeida-Paes R., Do Valle A.C.F. (2019). Clinical features and genetic background of the sympatric species *Paracoccidioides brasiliensis* and *Paracoccidioides americana*. PLoS Negl. Trop. Dis..

[66] Olsen S.K., Capili A.D., Lu X., Tan D.S., Lima C.D. (2010). Active site remodelling accompanies thioester bond formation in the SUMO E1. Nature.

[67] Mayer F.L., Sánchez-León E., Kronstad J.W. (2018). A chemical genetic screen reveals a role for proteostasis in capsule and biofilm formation by *Cryptococcus neoformans*. Microb. Cell.

[68] Szewczyk E., Chiang Y.M., Oakley C.E., Davidson A.D., Wang C.C.C., Oakley B.R. (2008). Identification and characterization of the asperthecin gene cluster of *Aspergillus nidulans*. Appl. Environ. Microbiol..

[69] Harting R., Bayram Ö., Laubinger K., Valerius O., Braus G.H. (2013). Interplay of the fungal sumoylation network for control of multicellular development. Mol. Microbiol..

[70] Horio T., Szewczyk E., Oakley C.E., Osmani A.H., Osmani S.A., Oakley B.R. (2019). SUMOlock reveals a more complete *Aspergillus nidulans* SUMOylome. Fungal Genet. Biol..

[71] Johnson E.S., Blobel G. (1999). Cell cycle-regulated attachment of the ubiquitin-related protein SUMO to the yeast septins. J. Cell Biol..

[72] Takahashi Y., Iwase M., Konishi M., Tanaka M., Toh-e A., Kikuchi Y. (1999). Smt3, a SUMO-1 homolog, is conjugated to Cdc3, a component of septin rings at the mother-bud neck in budding yeast. Biochem. Biophys. Res. Commun..

[73] Christmann M., Schmaler T., Gordon C., Huang X., Bayram Ö., Schinke J., Stumpf S., Dubiel W., Braus G.H. (2013). Control of multicellular development by the physically interacting deneddylases DEN1/DenA and COP9 signalosome. PLoS Genet..

[74] Mukhopadhyay D., Dasso M. (2007). Modification in reverse: The SUMO proteases. Trends Biochem. Sci..

[75] Pfaller M.A., Diekema D.J., Turnidge J.D., Castanheira M., Jones R.N. (2019). Twenty years of the SENTRY antifungal surveillance program: Results for *Candida* species from 1997–2016. Open Forum Infect. Dis..

[76] Pfaller M.A., Andes D.R., Diekema D.J., Horn D.L., Reboli A.C., Rotstein C., Franks B., Azie N.E. (2014). Epidemiology and outcomes of invasive candidiasis due to non-albicans species of *Candida* in 2496 patients: Data from the Prospective Antifungal Therapy (PATH) registry 2004–2008. PLoS ONE.

[77] Montagna M.T., Lovero G., Borghi E., Amato G., Andreoni S., Campion L., Lo Cascio G., Lombardi G., Luzzaro F., Manso E. (2014). Candidemia in intensive care unit: A nationwide prospective observational survey (GISIA-3 study) and review of the European literature from 2000 through 2013. Eur. Rev. Med. Pharmacol. Sci..

[78] Chakrabarti A., Sood P., Rudramurthy S.M., Chen S., Kaur H., Capoor M., Chhina D., Rao R., Eshwara V.K., Xess I. (2015). Incidence, characteristics and outcome of ICU-acquired candidemia in India. Intensive Care Med..

[79] Astvad K.M.T., Johansen H.K., Røder B.L., Rosenvinge F.S., Knudsen J.D., Lemming L., Schønheyder H.C., Hare R.K., Kristensen L., Nielsen L. (2018). Update from a 12-year nationwide fungemia surveillance: Increasing intrinsic and acquired resistance causes concern. J. Clin. Microbiol..

[80] Jones T., Federspiel N.A., Chibana H., Dungan J., Kalman S., Magee B.B., Newport G., Thorstenson Y.R., Agabian N., Magee P.T. (2004). The diploid genome sequence of Candida albicans. Proc. Natl. Acad. Sci. USA.

[81] Mayer F.L., Wilson D., Hube B. (2013). *Candida albicans* pathogenicity mechanisms. Virulence.

[82] Galocha M., Pais P., Cavalheiro M., Pereira D., Viana R., Teixeira M.C. (2019). Divergent approaches to virulence in *C. albicans* and *C. glabrata*: Two sides of the same coin. Int. J. Mol. Sci..

[83] Dujon B., Sherman D., Fischer G., Durrens P., Casaregela S., Lafentaine I., De Montigny J., Marck C., Neuvéglise C., Talla E. (2004). Genome evolution in yeasts. Nature.

[84] Kaur R., Domergue R., Zupancic M.L., Cormack B.P. (2005). A yeast by any other name: *Candida glabrata* and its interaction with the host. Curr. Opin. Microbiol..

[85] Bolotin-Fukuhara M., Fairhead C. (2014). *Candida glabrata*: A deadly companion?. Yeast.

[86] Kumar K., Askari F., Sahu M.S., Kaur R. (2019). *Candida glabrata*: A lot more than meets the eye. Microorganisms.

[87] De Groot P.W.J., Bader O., de Boer A.D., Weig M., Chauhan N. (2013). Adhesins in human fungal pathogens: Glue with plenty of stick. Eukaryot. Cell.

[88] Kaur R., Ma B., Cormack B.P. (2007). A family of glycosylphosphatidylinositol-linked aspartyl proteases is required for virulence of *Candida glabrata*. Proc. Natl. Acad. Sci..

[89] Cuéllar-Cruz M., Briones-Martin-del-Campo M., Cañas-Villamar I., Montalvo-Arredondo J., Riego-Ruiz L., Castaño I., De Las Peñas A. (2008). High resistance to oxidative stress in the fungal pathogen *Candida glabrata* is mediated by a single catalase, Cta1p, and is controlled by the transcription factors Yap1p, Skn7p, Msn2p, and Msn4p. Eukaryot. Cell.

[90] Leach M.D., Brown A.J.P. (2012). Posttranslational modifications of proteins in the pathobiology of medically relevant fungi. Eukaryot. Cell.

[91] Yan M., Nie X., Wang H., Gao N., Liu H., Chen J. (2015). SUMOylation of Wor1 by a novel SUMO E3 ligase controls cell fate in *Candida albicans*. Mol. Microbiol..

[92] Islam A., Tebbji F., Mallick J., Regan H., Dumeaux V., Omran R.P., Whiteway M. (2019). Mms21: A putative SUMO E3 ligase in *Candida albicans* that negatively regulates invasiveness and filamentation, and is required for the genotoxic and cellular stress response. Genetics.

[93] Huaping L., Jie L., Zhifeng W., Yingchang Z., Yuhuan L. (2007). Cloning and functional expression of ubiquitin-like protein specific proteases genes from *Candida albicans*. Biol. Pharm. Bull..

[94] Omeara T.R., Veri A.O., Ketela T., Jiang B., Roemer T., Cowen L.E. (2015). Global analysis of fungal morphology exposes mechanisms of host cell escape. Nat. Commun..

[95] Martin S.W., Konopka J.B. (2004). SUMO modification of septin-interacting proteins in *Candida albicans*. J. Biol. Chem..

[96] Conrad K.A., Rodriguez R., Salcedo E.C., Rauceo J.M. (2018). The *Candida albicans* stress response gene Stomatin-Like Protein 3 is implicated in ROS-induced apoptotic-like death of yeast phase cells. PLoS ONE.

